# AIDS Therapy, 3rd Edition

**DOI:** 10.3201/eid1405.080116

**Published:** 2008-05

**Authors:** David Rimland

**Affiliations:** * Veterans Administration Medical Center, Decatur, Georgia, USA

Reviewing and summarizing the treatment of HIV disease and its complications is a daunting task. Writing a textbook incorporating the rapidly evolving treatments and management strategies is even more difficult. In this third edition of AIDS Therapy, the authors have combined the efforts of international experts to fulfill this goal. As with every textbook, references are a little outdated; few references are more recent than 2006. The addition of online access to updates will possibly alleviate this problem, although the online version still lists the Department of Health and Human Services guidelines for antiretroviral use from October 2006.

Excellent chapters cover the serologic diagnosis of HIV disease, primary care in industrialized and resource-limited countries, strategic use of antiretroviral agents, immune-based therapies, and special clinical settings. Although the management of pregnant HIV-positive patients is discussed, no individual coverage of pediatrics is provided.

The text provides comprehensive reviews of each antiretroviral agent, summarizing pharmacology, adverse reactions, and clinical uses, and extensively reviewing major trials for each agent. For some of these agents, this represents a historical review of monotherapy without practical application. For example, a full chapter is devoted to zalcitabine, an agent that was discontinued in June 2006. For antiretroviral agents, the best summary, referred to as “recommendations for use,” is included in the last section of each drug chapter. 

Individual chapters describe opportunistic infections and malignancies, including their diagnosis, therapy, and prevention of these diseases. Variability in the length of these chapters does not always correlate with the importance of these processes. The inclusion of multiple charts and algorithms provides a useful approach to diagnosis and management. The last major section of the text provides approaches to specific syndromes including the major problems in patient care. These are excellent chapters and will be useful to clinicians evaluating specific syndromes. The lack of color pictures in the dermatologic and oral manifestations sections (even in the online version) is a drawback. The final chapters on drug administration and medications are useful tabulations of drug interactions, dosing, and adverse events.

This is an excellent comprehensive source book for AIDS clinicians, although it should not be considered a rapid guide to treatment options. This is a text that will be useful for understanding the basis of our current drug therapy. In contrast, the chapters discussing specific disease processes or syndromes will be extremely useful for the busy clinician looking for a single source for these conditions.

